# Are population‐based patient‐reported outcomes associated with overall survival in patients with advanced pancreatic cancer?

**DOI:** 10.1002/cam4.2704

**Published:** 2019-11-17

**Authors:** Wei Fang Dai, Jaclyn Beca, Helen Guo, Wanrudee Isaranawatchai, Deborah Schwartz, Rohini Naipaul, Jessica Arias, Yao Qiao, Scott Gavura, Ruby Redmond‐Misner, Zahra Ismail, Lisa Barbera, Kelvin Chan

**Affiliations:** ^1^ Cancer Care Ontario Toronto ON Canada; ^2^ Canadian Centre for Applied Research in Cancer Control Toronto ON Canada; ^3^ St Michael's Hospital Toronto ON Canada; ^4^ Insitute of Health Policy, Management and Evaluation University of Toronto Toronto ON Canada; ^5^ Tom Baker Cancer Centre Calgary AB Canada; ^6^ University of Calgary Calgary AB Canada; ^7^ Sunnybrook Health Sciences Centre Toronto ON Canada

**Keywords:** advanced pancreatic cancer, patient‐reported outcome measures, survival

## Abstract

**Background:**

Advanced pancreatic cancer (APC) patients often have substantial symptom burden. In Ontario, patients routinely complete the Edmonton Symptom Assessment Scale (ESAS), which screens for nine symptoms (scale: 0‐10), in cancer clinics. We explored the association between baseline patient‐reported outcomes, via ESAS, and overall survival (OS).

**Methods:**

Advanced pancreatic cancer patients with ESAS records prior to receiving publicly funded drugs from November 2008 to March 2016 were retrospectively identified from Cancer Care Ontario's administrative databases. We examined three composite ESAS scores: total symptom distress score (TSDS: 9 symptoms), physical symptom score (PHS: 6/9 symptoms), and psychological symptom score (PSS: 2/9 symptoms); Composite scores greater than defined thresholds (TSDS ≥36, PHS ≥24, PSS ≥8) were considered as high symptom burden. Crude OS was assessed using Kaplan‐Meier method. Hazard ratios (HRs) were assessed using multivariable Cox models. Analysis was repeated in a sub‐cohort with Eastern Cooperative Oncology Group (ECOG) status and metastasis.

**Results:**

We identified 2199 APC patients (mean age 64 years, 55% male) with ESAS records prior to receiving chemotherapy. Crude median survival was 4.5 and 7.3 months for high and low TSDS, respectively. High TSDS was associated with lower OS (HR = 1.47, 95% CI: 1.33, 1.63). In the sub‐cohort (n = 393) with ECOG status and metastasis, high TSDS was also associated with lower OS (HR = 1.34, 95% CI: 1.04, 1.73). Similar trends were observed for PHS and PSS.

**Conclusions:**

Higher burden of patient‐reported outcome was associated with reduced OS among APC patients. The effect was prominent after adjusting for ECOG status.

## Graphical abstract

In a cohort of advanced pancreatic cancer patients, we observed that higher burden of patient‐reported symptoms at baseline is associated with reduced overall survival. The association remained significant after accounting for performance status and type of treatment.

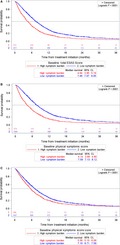

## 1 INTRODUCTION

Pancreatic cancer is the seventh leading cause of cancer‐related mortality and 11th most common cancer globally.^1^ At diagnosis, majority of patients with advanced pancreatic cancer (APC) are present with either locally advanced or metastatic pancreatic adenocarcinoma with median survival of 9‐15 months and 3‐6 months, respectively.^2^ APC patients are primarily managed with systemic chemotherapy that aims to prolong survival and improve or maintain the quality of life. While standard chemotherapy treatments for APC have evolved from single‐agent gemcitabine therapy to include more active combination therapies, APC diagnosis still confers poor prognosis.^3^, ^4^

Well‐established prognostic factors in pancreatic cancer include clinical and pathological stage, performance status (PS), surgical margins, and biomarkers. PS has also shown significant association with survival among patients treated with gemcitabine, irrespective of disease stage.^5^, ^6^, ^7^, ^8^ Previous studies have shown that advanced cancer patients randomized to routine symptom reporting have better survival outcomes.^9^ However, there is a paucity of literature exploring the association between patient‐reported outcomes, independent of PS, with survival outcome in the real‐world.

Patients with APC often report high symptom burden and one of the main intents of systemic chemotherapy is to palliate these symptoms. In a previous study, 95% patients reported at least one of the 13 symptoms from MD Anderson Symptom Inventory at initial assessment.^10^ An integrative review of 16 studies found that at least 25% of patients with APC reported moderate to severe physical (fatigue, loss of appetite, pain, insomnia, and digestive symptoms) or psychological (impaired well‐being, anxiety, fear, and depression) symptom burden.^11^ The most common and distressing symptoms reported from patients include fatigue, loss of appetite, and pain.^11^, ^12^

In 2007, Cancer Care Ontario, Ontario's provincial cancer agency, began to implement routine screening with the Edmonton Symptoms Assessment System (ESAS) for cancer patients.^13^, ^14^, ^15^ ESAS is a validated and reliable patient‐reported outcomes measure designed to assess symptoms in palliative cancer populations.^16^ The Ontario Cancer Symptoms Management Collaborative (OCSMC) was subsequently established and expanded the program to electronic systematic symptom screening, using the Interactive Symptoms Assessment and Collection (ISAAC) system, for all cancer patients in the 14 regional cancer centers and their partner hospitals in Ontario.^13^, ^14^, ^15^, ^16^ The province aim to screen 70% of the ambulatory oncology patients seen at the regional cancer center at least once each month.^15^ In 2013, Patient‐Reported Functional Status, a version of Eastern Cooperative Oncology Group (ECOG) PS, was added to ISAAC to supplement ESAS.^15^ Currently, over 30 000 unique patients report their symptoms via ISAAC every month, and the Symptom Management database has over 6.5 million symptom assessments.^14^ This large‐scale, standardized symptom assessment program provides a unique opportunity to understand the impact of patient‐reported outcomes in clinical care.

For a cancer that progresses rapidly with high symptom burden such as APC, symptoms reported by patients at presentation can be a prognostic factor for patient outcomes. In this study, we investigated the association between baseline symptoms, as measured by ESAS, and overall survival (OS) in patients with APC.

## 2 MATERIALS AND METHODS

### 2.1 Study design

A population‐based retrospective cohort study was performed using APC patients from Ontario, Canada. Patients diagnosed with incident APC (ICD10‐25) from November 2008 to March 2016 with valid health insurance number were identified from Ontario Cancer Registry (OCR). The cohort was linked to the new drug funding program (NDFP) claims database to identify those who received treatment with one of the following publicly funded APC regimens: gemcitabine, FOLFIRINOX (leucovorin, fluorouracil, irinotecan, and oxaliplatin), or gemcitabine with nab‐paclitaxel.

Subsequently, the cohort was linked to the Symptoms Management database to obtain ESAS records and other administrative databases, including Canadian Institute for Health Information's Discharge Abstract Database (CIHI‐DAD) and activity level reporting (ALR) database, to extract baseline characteristics and outcomes. Patients were included if they had an ESAS record prior to receiving publicly funded drugs for APC. Patients were excluded if the ESAS assessments were incomplete, or no baseline ESAS assessment was collected within 60 days before their treatment start date.

#### 2.1.1 Outcomes

The primary outcome, OS, was measured from the start date of chemotherapy, as captured in the NDFP, to the time of death due to any cause as captured in the OCR or until the last date of follow‐up, 30 May 2016.

#### 2.1.2 Patient‐reported symptoms—ESAS

Edmonton Symptom Assessment Scale consists of nine common symptoms including pain, tiredness, nausea, depression, anxiety, drowsiness, loss of appetite, well‐being, and shortness of breath.^17^ For each symptom, patients report the severity at the time of assessment on a scale from 0 (absence of symptom) to 10 (worst possible symptom).^17^ Patients typically complete ESAS during their visit to the cancer center or hospital; patients have the option to complete ESAS at home, few patients use this option.

Edmonton Symptom Assessment Scale symptom severity can be categorized as absent (0), mild (1‐3), moderate (4‐6), or severe (7‐10).^18^, ^19^, ^20^, ^21^ Scores greater than 4 were considered clinically meaningful.^18^, ^19^ All nine ESAS symptoms can be summed together to determine the total symptom distress score (TSDS). In addition, individual ESAS symptoms can be grouped into a composite physical score (PHS), including pain, tiredness, nausea, drowsiness, appetite, and shortness of breath, and a composite psychological score (PSS), including depression and anxiety.^20^, ^22^ A priori, we dichotomized patient's baseline ESAS score into high or low symptom burden by multiplying the clinically meaningful threshold of four by the number of symptoms in the aggregated composite score. Patients are categorized as high symptom burden if they had scored above the clinically meaningful threshold for TSDS (≥36/90), PHS (≥24/60), or PHS (≥8/20), otherwise they are categorized as low symptom burden.

#### 2.1.3 Covariates

Baseline characteristics including age at first treatment and gender were identified. Postal code from the OCR was used to determine neighborhood income quintile (linked by patient's postal code to Statistics Canada 2006 Census data), urban residence, and region. Clinical characteristics such as cancer diagnosis, diagnosis date, and death date were obtained from the OCR. First‐line treatment, metastasis (vs locally advanced), ECOG PS scores for selected patients, and treatment date were obtained from the NDFP claims database. Previous pancreatic cancer resections were extracted from CIHI‐DAD. ALR was used to identify patients who received prior adjuvant gemcitabine or radiation therapy. Charlson‐Deyo score was calculated for 2 years prior to the first treatment based on the diagnosis code in CIHI‐DAD.

### 2.2 Statistical analysis

Descriptive statistics were calculated to examine the summary of study covariates overall and by baseline ESAS severity. Baseline differences for categorical variables, presented as percentages, were calculated using Pearson Chi‐square test. Comparison between continuous variables, presented as mean and standard deviation, was calculated using one‐way ANOVA.

**Figure 1:**
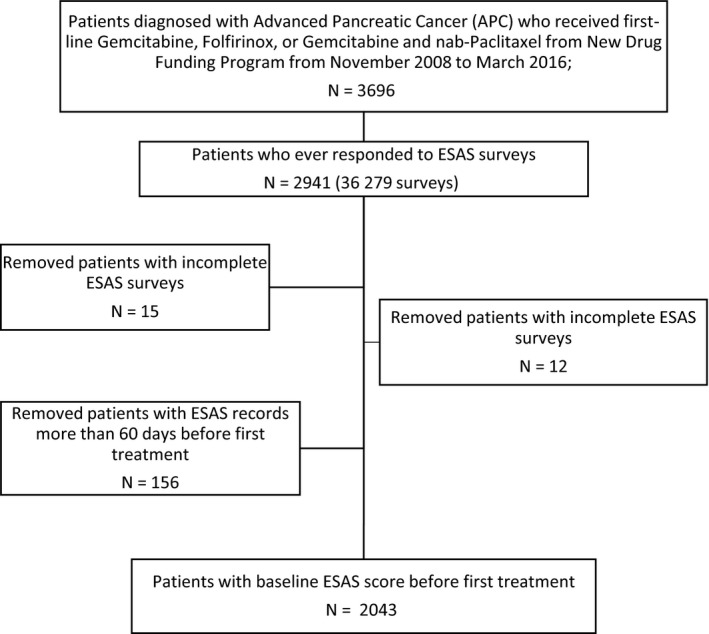
Study cohort creation. Acronyms: ESAS, Edmonton Symptom Assessment Scale; RPDB, registered persons database; OCR, Ontario Cancer Registry.

Overall survival was estimated using Kaplan‐Meier method and subgroups (high vs low symptom burden) were compared using the Log‐rank test. Univariable Cox proportional hazard models were conducted to calculate hazard ratios (HR) and respective 95% confidence intervals (CI). To quantify the effect of each baseline ESAS composite score on OS in the presence of other covariates, multivariable Cox proportional hazard models were also performed. As a sensitivity analysis, continuous baseline ESAS scores were categorized as mild, moderate, and severe. Survival analysis was repeated for the tertiary categorization (Figure 1 in Appendix S1 and Table 3 in Appendix S2). Additional sensitivity analysis was conducted to assess different cutoff threshold for composite ESAS score and “look‐back” time window definition for baseline ESAS (Table 8 & Table 9 in Appendix S2). In a sub‐cohort of patients with data on ECOG PS and metastasis, multivariable Cox proportional hazards model was used to evaluate the effects of these potential confounders on OS.

Proportional hazards assumptions were assessed. All calculations were performed with SAS software (version 9.4; SAS Institute). The significance level was set to less than .05 for all two‐sided *P*‐values.

## 3 RESULTS

### 3.1 Cohort characteristics

A total of 2043 APC patients with baseline ESAS records were included in the analysis (Figure 1). On average, patients were 65 years old (SD: 10.0), male (55%), and most lived in urban regions (85%) (Table 1). The majority of patients in the cohort received either gemcitabine (54%) or FOLFIRINOX (40%) as their first‐line chemotherapy and the average duration of treatment was 5.75 months (SD: 5.77). The median follow‐up time for the overall cohort was 9.2 months (SD: 5.8). In the total cohort, 62% of patients died within the first year of treatment and 88% died prior to the end of the follow‐up period.

**Table 1:** Baseline characteristics

	Total	Total symptom distress scores (TSDS)		*P*‐value	Physical symptom scores (PHS)		*P*‐value	Psychological symptom scores (PSS)		*P*‐value
		Low (0‐35) n = 1471	High (36‐90) n = 572		Low (0‐23) n = 1654	High (24‐60) n = 545		Low (0‐7) n = 1664	High (8‐20) n = 535	
Age, mean ± SD	65.0 ± 10.0	65.3 ± 9.9	64.4 ± 10.3	.007	65.4 ± 9.8	64.0 ± 10.4	.006	65.3 ± 9.94	64.4 ± 10.1	.07
Male, n (%)	1123 (55.0)	836 (56.8)	287 (50.2)	.007	835 (56.8)	288 (50.2)	.007	819 (56.6)	304 (51.0)	.02
Income quintile, n (%)										
Lowest	316 (15.5)	215 (14.6)	101 (17.6)	.04	215 (14.6)	101 (17.6)	.002	209 (14.4)	107 (17.9)	.01
Medium to low	346 (16.9)	249 (16.9)	97 (17.0)		252 (17.2)	94 (16.4)		236 (16.3)	110 (18.5)	
Middle	373 (18.3)	268 (18.2)	105 (18.4)		277 (18.9)	96 (16.7)		270 (18.7)	103 (17.3)	
Medium to high	408 (20.0)	301 (20.5)	107 (18.7)		293 (20.0)	115 (20.0)		292 (20.2)	116 (19.5)	
Highest	427 (20.9)	326 (22.2)	101 (17.6)		327 (22.3)	100 (17.4)		327 (22.6)	100 (16.8)	
Unknown	173 (8.4)	112 (7.6)	61 (10.7)		105 (7.2)	68 (11.9)		113 (7.8)	60 (10.1)	
Rurality, n (%)										
Urban	1745 (85.4)	1251 (85.0)	494 (86.4)	.74	1255 (85.4)	490 (85.3)	.99	1218 (84.2)	527 (88.4)	.04
Rural	280 (13.7)	207 (14.1)	73 (12.8)		201 (13.7)	79 (13.8)		214 (14.8)	66 (11.1)	
Unknown	18 (0.9)	13 (0.9)	5 (0.8)		13 (0.9)	5 (0.9)		15 (1.0)	<5	
Charlson‐Deyo Score, n (%)										
0	1339 (65.5)	934 (63.5)	405 (70.8)	.001	933 (63.5)	406 (70.7)	.0001	921 (63.7)	418 (70.1)	<.001
1	47 (2.3)	30 (2.0)	17 (3.0)		27 (1.8)	20 (3.5)		31 (2.1)	16 (2.7)	
2+	657 (32.2)	507 (34.5)	150 (26.2)		509 (34.7)	148 (25.8)		495 (34.2)	162 (27.2)	
Previous radiation, n (%)	311 (15.2)	234 (15.9)	77 (13.5)	.17	226 (15.4)	85 (14.8)	.75	228 (15.8)	83 (13.9)	.30
Previous pancreatic resection, n (%)	383 (18.8)	309 (21.0)	74 (12.9)	<.001	318 (21.7)	65 (11.3)	<.001	300 (20.7)	83 (18.9)	<.001
Previous adjuvant gemcitabine, n (%)	120 (5.9)	98 (6.6)	22 (3.9)	.015	100 (6.8)	20 (3.5)	.004	88 (6.1)	32 (5.4)	.5
Treatment regimen, n (%)										
Gemcitabine	1103 (54.0)	757 (51.5)	346 (60.5)	.001	759 (51.7)	344 (59.9)	.002	767 (53.0)	336 (56.4)	.14
FOLFIRINOX	809 (39.6)	621 (42.2)	188 (32.9)		617 (42.0)	192 (33.5)		592 (40.9)	217 (36.4)	
Gemcitabine nab‐paclitaxel	131 (6.4)	93 (6.3)	38 (6.6)		93 (6.3)	38 (6.6)		88 (4.31)	43 (7.21)	
Follow‐up (months), mean ± SD	9.2 ± 9.8	10.1 ± 10.2	6.9 ± 8.1	<.001	10.2 ± 10.2	6.6 ± 8.1	<.001	9.6 ± 10.0	8.0 ± 9.1	<.001
First‐line duration (months), mean ± SD	4.5 ± 5.3	5.0 ± 5.6	3.3 ± 4.2	<.001	5.0 ± 5.5	3.3 ± 4.3	<.001	4.8 ± 5.5	3.9 ± 4.7	<.001
Time to first‐line treatment (mo), mean	4.8 ± 0.22	4.9 ± 0.3	4.5 ± 0.4	.40	5.0 ± 0.3	4.3 ± 0.4	.15	4.6 ± 0.2	5.1 ± 0.5	.4

Abbreviation: SD, standard deviation.

Table 1 shows the baseline characteristics by symptom burden. Patients with low total symptom burden at baseline were more likely to be older, male, and in the highest income quintile. They also tended to have higher Charlson‐Deyo score, have had previous pancreatic resection, or received previous adjuvant gemcitabine. Similar trends were observed for physical and psychological symptom burdens. Patients who reported higher total or physical symptom burden at baseline were more likely to receive gemcitabine, whereas those who reported low symptom burden were more likely to receive FOLFIRINOX.

At baseline, majority of patients reported low total (72%), physical (72%), and psychological (71%) symptom burden (Table 1 in Appendix S2). The average score for TSDS, PHS, and PSS were 26.3 (SD: 17.1), 17.0 (SD: 11.8), and 5.2 (SD: 5.0), respectively (Table 1 in Appendix S2). Individual symptoms with average scores at or above the clinically significant threshold of 4.0 points included: Tiredness (4.2, 95% CI: 4.0, 4.3), loss of appetite (4.0, 95% CI: 3.9, 4.1), and general well‐being (4.0, 95% CI: 3.9, 4.1) (Table 2 in Appendix S2).

### 3.2 Survival outcomes

**Figure 2:**
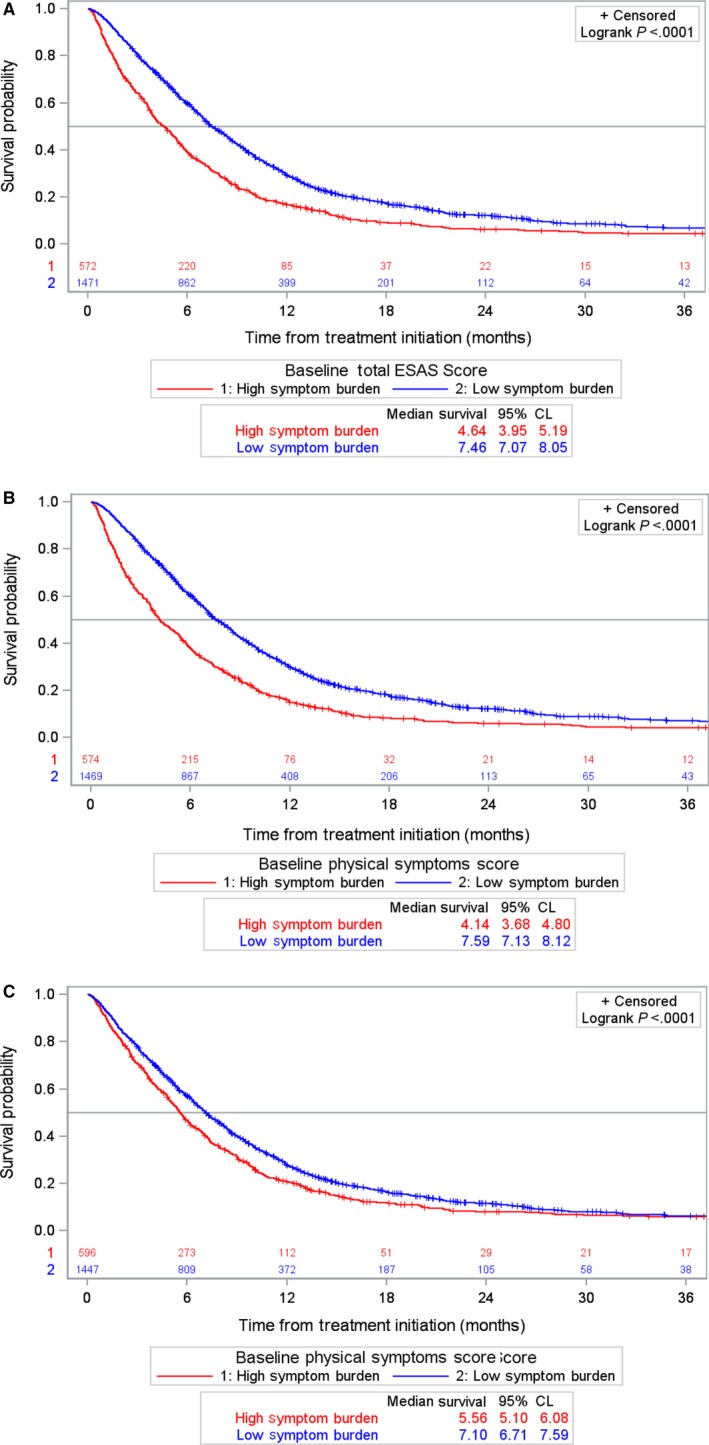
Kaplan‐Meier estimates of overall survival (OS) by baseline composite Edmonton Symptom Assessment Scale (ESAS) scores. OS in 2043 advanced pancreatic cancer patients who reported baseline composite ESAS scores prior to receiving first‐line chemotherapy treatment. A, Baseline composite total symptom distress scores (TSDS), (B) Baseline composite physical symptom scores (PHS), and (C) Baseline composite psychological symptom scores (PSS)

Patients who reported high TSDS at baseline experienced shorter OS compared to patients reporting low TSDS (median OS: 4.6 vs 7.5 months), with a crude HR of 1.54 (95% CI: 1.39, 1.70) (Figure 2A). Similar results were observed for baseline physical and psychological burdens (Figure 2B,C). Sensitivity analysis was conducted to assess different ESAS cutoff and different time period definition for baseline ESAS. We observed a robust association between high symptom burden and shorter OS (Tables 8 & 9 in Appendix S2).

Survival analysis was repeated among subgroups of patients by treatment regimen (Table 2). Among patients receiving gemcitabine, OS was significantly shorter for patients reporting high TSDS (median: 3.66 vs 6.81 months, HR = 1.57), PHS (median: 3.37 vs 6.94 months, HR = 1.71), and PSS (median: 4.95 vs 6.21 months, HR = 1.20) (Table 2). Similar associations between survival and baseline symptom burden were observed for patients receiving FOLFIRINOX and the smaller gemcitabine+nab‐paclitaxel group, but the latter associations were not statistically significant. Median OS estimate and HR by baseline covariates are presented in Table 6 in Appendix S2.

**Table 2:** Median survival and hazard ratio of baseline composite ESAS scores

	Median survival, mo (95% CI)		Crude hazard ratio (95% CI)
	High symptom burden	Low symptom burden	
Total cohort (n = 2034)			
TSDS	4.64 (3.95, 5.20)	7.46 (7.07, 8.06)	1.54 (1.39, 1.70)*
PHS	4.14 (3.68, 4.80)	7.60 (7.13, 8.12)	1.64 (1.48, 1.81)*
PSS	5.56 (5.10, 6.08)	7.10 (6.71, 7.60)	1.23 (1.11, 1.36)*
Gemcitabine (n = 1103)			
TSDS	3.66 (3.32, 4.21)	6.81 (6.21, 7.27)	1.57 (1.37, 1.78)*
PHS	3.37 (2.80, 3.81)	6.94 (6.35, 7.53)	1.71 (1.50, 1.95)*
PSS	4.95 (3.95, 5.56)	6.21 (5.49, 6.71)	1.20 (1.05, 1.37)*
Gemcitabine+nab‐paclitaxel (n = 131)			
TSDS	4.37 (2.00, 5.92)	6.18 (4.60, 7.30)	1.30 (0.86, 1.95)
PHS	3.65 (1.58, 5.92)	6.18 (4.67, 7.30)	1.28 (0.85, 1.93)
PSS	4.70 (3.26, 6.15)	6.15 (4.41, 7.43)	1.36 (0.92, 2.02)
FOLFIRINOX (n = 809)			
TSDS	6.12 (5.23, 7.04)	8.84 (8.19, 9.63)	1.44 (1.21, 1.72)*
PHS	6.31 (5.26, 7.20)	8.81 (8.19, 9.63)	1.51 (1.27, 1.81)*
PSS	6.97 (5.98, 7.96)	8.52 (7.86, 9.50)	1.25 (1.05, 1.48)*

Abbreviations: CI, confidence interval; TSDS, baseline total symptom distress score; PHS, baseline physical symptom scores; PSS, baseline psychological symptom scores.

* *P* < .05.

For each individual symptom score, we explored changes in OS by the gradient of ESAS score: absent (score = 0), mild (<4), moderate (4‐6), and severe (>7) (Table 3 in Appendix S2). We observed that patients who reported severe baseline symptom scores have the shortest median OS, and those who reported absent baseline symptom scores have the longest median OS. Patients who reported severe baseline drowsiness and shortness of breath had the shortest median OS, while those who reported absent lack of appetite and best general well‐being at baseline had the longest median OS.

Multivariable Cox proportional hazards regression models were used to adjust for potential covariates (Table 3). After adjusting for baseline characteristics and treatment regimen, higher baseline TSDS was associated with worse OS (HR = 1.50, 95% CI: 1.35, 1.66). Moreover, FOLFIRINOX was associated with lower mortality (HR = 0.75, 95% CI: 0.67, 0.83) and gemcitabine+nab‐paclitaxel (HR = 1.24, 95% CI: 1.02, 1.51) was associated with higher mortality when compared to gemcitabine. Similarly, baseline PHS was associated with OS after adjustment (HR = 1.59, 95% CI: 1.43, 1.77). While baseline PSS adjusting for baseline characteristics was associated with OS (HR = 1.20, 95% CI: 1.08, 1.32), the effect of PSS (*p* = .79) was no longer observed after adjusting for both baseline PHS and PSS. When the ESAS score was modeled as an ordinal variable (mild, moderate or severe), similar results were observed (Table 4 in Appendix S2).

**Table 3:** Multivariable Cox proportional hazard ratio estimates adjusting for baseline composite ESAS symptom burden

	Model 1^a^	Model 2^a^	Model 3^a^	Model 4^a^
	HR (95% CI)	HR (95% CI)	HR (95% CI)	HR (95% CI)
TSDS				
High vs low	1.50 (1.35, 1.66)*	—	—	—
PHS				
High vs low	—	1.59 (1.43, 1.77)*	—	1.58 (1.41, 1.78)*
PSS				
High vs low	—	—	1.20 (1.08, 1.32)*	1.02 (0.91, 1.14)
Treatment				
FOLFIRINOX vs gemcitabine	0.75 (0.67, 0.83)*	0.75 (0.67, 0.83)*	0.73 (0.66, 0.82)*	0.75 (0.67, 0.83)*
Gemcitabine‐nab paclitaxel vs gemcitabine	1.24 (1.02, 1.51)*	1.23 (1.01, 1.50)*	1.24 (1.01, 1.51)*	1.23 (1.01, 1.50)*

Abbreviations: CI, confidence interval; TSDS, baseline total symptom distress score; PHS, baseline physical symptom scores; PSS, baseline psychological symptom scores.

a Model also adjusted for age at first‐line treatment, gender, rurality, neighborhood income quintile, Charlson‐Deyo score, previous adjuvant gemcitabine, previous pancreatic resection, and previous radiation.

* *P* < .05.

In a sub‐cohort of 367 patients, we performed a sensitivity analysis by adjusting for baseline ECOG PS and metastasis in each of the models specified above (Table 5 in Appendix S2). After adjusting for ECOG PS and metastasis, both TSDS (HR: 1.42, 95% CI: 1.09, 1.85) and baseline PHS (HR: 1.57, 95% CI: 1.21, 2.10) continued to be associated with OS but baseline PSS was no longer significant (*P* = .57). Baseline PSS was also no longer significant (*P* = .53) when adjusting for PHS, PSS, ECOG PS, and metastasis.

## 4 DISCUSSION

In a population‐based study of 2043 patients with APC, we assessed the association between baseline patient‐reported outcomes (ESAS scores) and survival. We observed shorter OS among APC patients who reported higher symptom burden as measured by the total ESAS score, even after adjusting for baseline characteristics and treatment regimen. The effect of total ESAS score, containing both physical and psychological symptoms, can be mainly attributed to the composite physical symptoms. Additionally, individual patient‐reported symptoms were also significantly associated with OS. Furthermore, the association between patient‐reported symptoms and OS remained significant even after adjusting for ECOG PS and metastasis.

Given that there is paucity of literature examining different thresholds for composite ESAS scores, we conducted sensitivity analyses using different thresholds and “look‐back” window for baseline ESAS accrual. Our findings are consistently robust with the different definitions. We also observed that patients with previous pancreatic resection or adjuvant gemcitabine treatments were more likely to have reported lower symptom burdens prior to treatment. In particular, these patients reported significantly lower pain scores (mean: 2.35 vs 3.34; *P* < .01) prior to treatment. We hypothesize that patients who did not undergo pancreatic resection may have been experiencing more pain from the celiac plexus based on the location of the primary tumor.

While PS assessments have been shown to be prognostic for APC patients, the role of patient‐reported outcomes has rarely been explored. In 462 patients with non‐small cell lung cancer (NSCLC), baseline ESAS assessment as a prognostic tool for treatment choice and survival was studied and similar trends in median OS were observed^23^; however, the effect of baseline ESAS on survival was no longer significant after multivariable adjustment, which included PS.^23^ It is notable that the HR (HR = 1.50; 95% CI: 1.36‐1.66) for higher total ESAS score in our cohort of APC patients was comparable to the HR (HR = 1.78; 95% CI: 1.45‐2.18) for high total ESAS score among NSCLC patients.^23^ In another single‐center cohort study on patients with metastatic renal cell carcinoma, the HR for OS was 1.27 (95% CI: 1.08‐1.50) for every 10‐unit increase in total ESAS score.^24^

We observed that the association between baseline psychological symptoms and survival was no longer significant after adjusting for baseline physical symptoms. In contrast, existing literature has shown that psychological symptoms, such as depression, impact mortality in various cancers.^25^, ^26^, ^27^ Some researchers have suggested this effect might be explained by lower motivation in depressed patients to receive treatment. This may potentially explain the discrepancy with our results, as only treated patients were included in our study.

The results of this study should be interpreted in the context of several limitations. First, some prognostic factors, such as pathological characteristics, laboratory markers, and molecular markers, are not readily available in the administrative databases; hence they were not adjusted for in the multivariable models. The observed association between patient‐reported symptoms and OS may be biased if there is an association between the unobserved prognostic factors and baseline patient‐reported symptoms; however, the association was not observed in the existing literature. Second, ECOG PS was only available for a small cohort of the patients, which limits the interpretation of the independent effects of ECOG PS and ESAS symptoms on survival if the sub‐cohort with ECOG PS was not representative of the population. Given the introduction of ECOG PS data collection was an administrative change, there is no reason to suggest the effect would not be consistent in the larger cohort. Our study is also limited by the humanistic nature of ESAS collection, ESAS collection is both opportunistic and voluntary, and certain patients are more likely to complete an ESAS assessment. We observed more men reporting with lower symptom burden at baseline; however, gender was not independently associated with survival. (Table 6 in Appendix S2). In the 20% of patients without baseline ESAS, we observed shorter follow‐up time and greater mortality, suggesting patients with poor prognosis may be less likely to complete an ESAS assessment regardless of their symptom burden. If these patients with missing baseline ESAS were more likely to report low symptom burden, this could potentially attenuate the observed association. Lastly, ESAS questionnaires were unable to differentiate whether the symptoms were disease‐related or comorbid.

While our results showed robust association between baseline ESAS severity and survival, it is important to acknowledge that we did not establish causality in light of the above limitations. In particular, symptom burden may be an underlying manifestation of unfavorable biology and addressing the symptoms may not improve survival. A potential future direction can explore whether palliative care for patients with high symptom burden would change the survival outcome. Nevertheless, in the absence of the ability to randomize patients by the severity of their symptoms, population‐based studies in the real‐world provide the highest level of evidence plausible. Nonetheless, the worsen survival outcomes among patients with higher symptom burden, irrespective of treatment regimen received, suggest reasonable evidence and support for using patient‐reported outcomes as a prognostic tool.

Patient‐reported outcome has also been suggested as a valuable tool for identifying symptoms from the patients who may be overlooked.^28^ As such, the collection of patient‐reported outcome prior to treatment can be a potential signal for early palliative and/or supportive care intervention, especially for patients with higher symptom burden. While clinicians may prescribe standard supportive and/or palliative care, when and whether these clinical services are provided may vary. In Ontario, where patients have complete coverage for outpatient clinic‐based and home‐based access to palliative care physician and nurses for symptom control, those who are routinely screened for ESAS were more likely to have palliative care initiated.^29^ Furthermore, studies have shown that patients receiving early initiation of palliative care have better survival outcomes than delayed initiation.^30^, ^31^, ^32^ While collecting patient‐reported outcomes may be resource intensive administratively, our results highlight the potential insights and benefits patient‐reported outcomes can provide for clinicians and patients. In particular, routine assessment of symptoms prior to treatment intiation may allow clinicians to incorporate supportive and palliative care into the treatment plan earlier on, which have been shown to deliver better clinical outcomes for patients.^30^, ^31^, ^32^

In conclusion, patients with APC who reported higher baseline symptom burden, as represented by moderate or severe ESAS scores, experienced reduced OS. The prognostic effect of baseline total ESAS score lies primarily with the physical symptoms. While our study demonstrates a possible clinical benefit for using ESAS as a prognostic tool, it is important to explore the change in ESAS score over treatment period and the health care utilization after treatment. Future investigation is required to understand the relationship between patient‐reported outcomes and OS in oncology setting and the impact of routine symptom screening on survival outcomes.

## CONFLICT OF INTEREST

The author(s) declare(s) no conflict of interest.

## AUTHOR CONTRIBUTIONS

Wei Fang Dai: Conceptualization, methodology, analysis, writing—original draft, reviewing, and editing; Jaclyn Beca: Conceptualization, methodology, writing—original draft, review, and editing; Helen Guo: Analysis and writing—review and editing; Wanrudee Isaranuwatchai: Methodology and writing—review and editing; Deborah Schwartz: Project administration and writing—review and editing; Rohini Naipaul: writing—review and editing; Jessica Arias: writing—review and editing; Yao Qiao: Analysis and writing—review and editing; Scott Gavura: writing—review and editing; Ruby Redmond‐Misner: Analysis, methodology, and writing—review and editing; Zahra Ismail: Writing—review and editing; Lisa Barbera: Writing—review and editing; Kelvin Chan: Conceptualization, methodology, writing—original draft, review, and editing

## Data Availability

The data that support the findings of this study are available from Cancer Care Ontario. Restrictions apply to the availability of these data, which were used under license for this study. Data are available from the authors with the permission of Cancer Care Ontario.
