# Supplementary material for: Are population‐based patient‐reported outcomes associated with overall survival in patients with advanced pancreatic cancer?
Source: Cancer Med. 2019 Nov 17;9(1):215–24. doi: 10.1002/cam4.2704 (PMC6943146; doi:10.1002/cam4.2704)
Supplement: Supplementary file 1 [file CAM4-9-215-s001.pdf]

Appendix Figure 1: Kaplan Meier Estimate of OS by Mild, Moderate and Severe Gradient of baseline composite ESAS scores

A

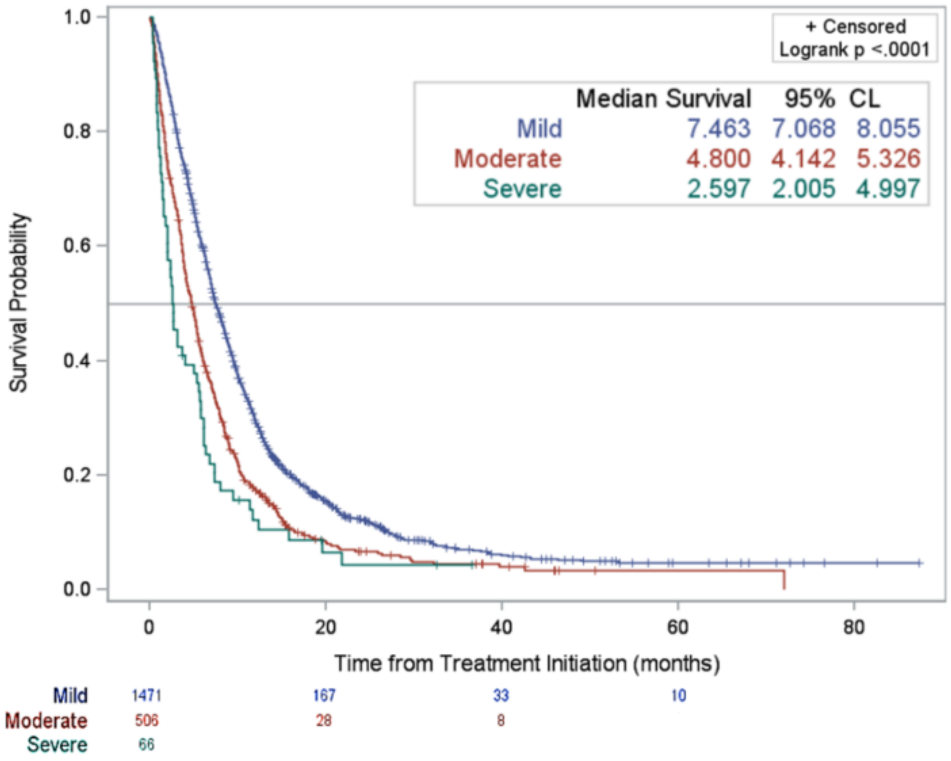

B

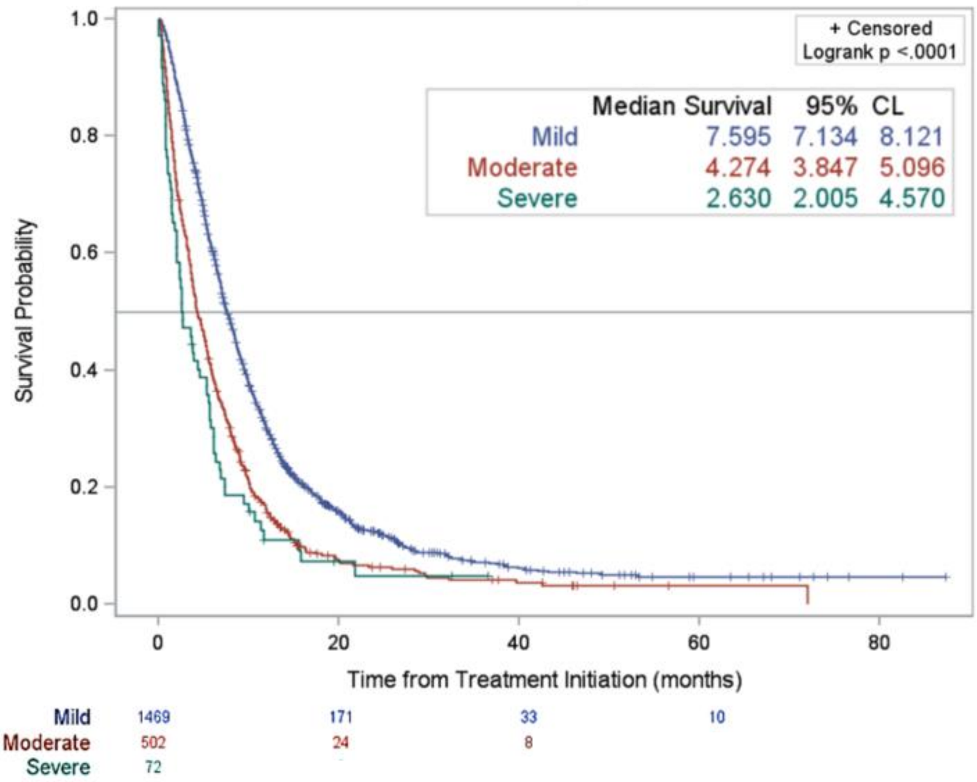

C

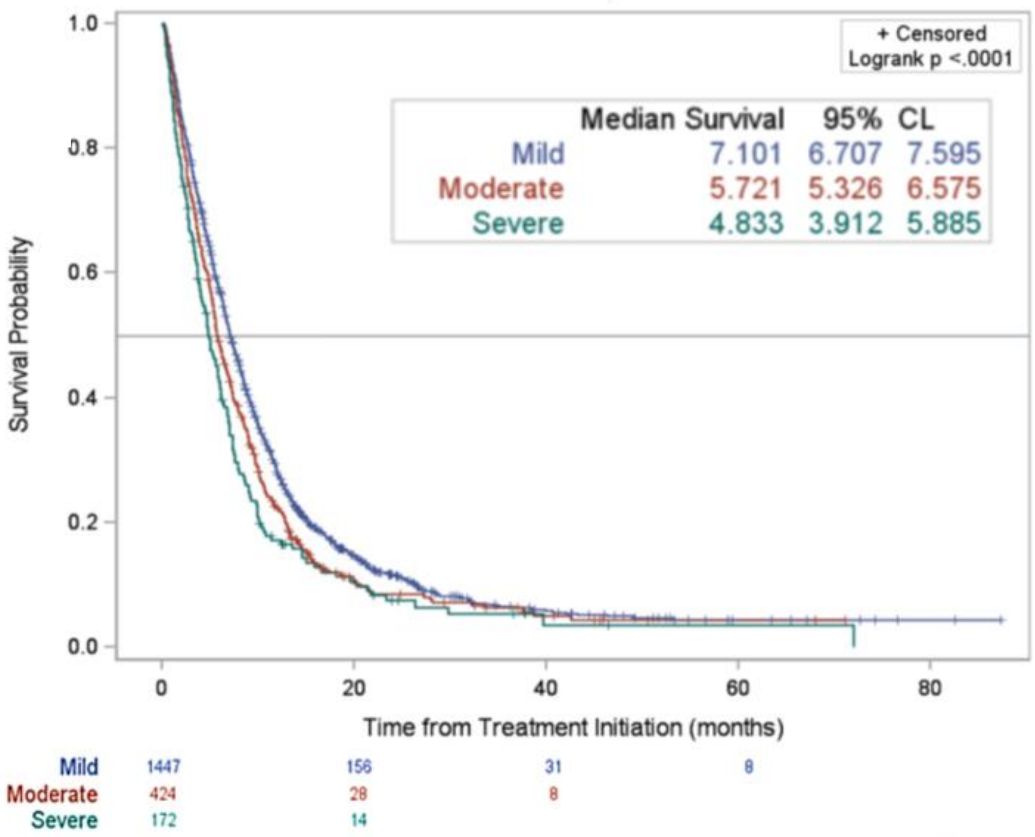
+ Censored
Logrank p <.0001
Median Survival 95% CL
Mild 7.101 6.707 7.595
Moderate 5.721 5.326 6.575
Severe 4.833 3.912 5.885
Survival Probability
1.0
0.8
0.6
0.4
0.2
0.0
0
20
40
60
80
Time from Treatment Initiation (months)
Mild 1447 156 31 8
Moderate 424 28 8
Severe 172 14
