# Supplementary material for: Are population‐based patient‐reported outcomes associated with overall survival in patients with advanced pancreatic cancer?
Source: Cancer Med. 2019 Nov 17;9(1):215–24. doi: 10.1002/cam4.2704 (PMC6943146; doi:10.1002/cam4.2704)
Supplement: Supplementary file 2 [file CAM4-9-215-s002.docx]

**Appendix:**

Appendix Table 1: Distribution of ESAS Burden Scores

|  | High Symptom Burden | Low Symptom Burden | Total |
| --- | --- | --- | --- |
|  |  |  |  |
| Total Symptom Distress Score (TSDS), n (%) | 572 (28%) | 1471 (72%) | 2043 |
| Total Symptom Distress Score (TSDS), mean (SD) | 48.38(10.27) | 17.65 (10.02) | 26.25(17.09) |
| Physical Symptom Score (PHS), n (%) | 574 (28.1%) | 1469 (71.9%) | 2043 |
| Physical Symptom Score (PHS), mean (SD) | 32.39 (7.12) | 11.03 (6.75) | 17.03 (11.80) |
| Psychological Symptom Score (PSS), n (%) | 596 (29.2%) | 1447 (70.8%) | 2043 |
| Psychological Symptom Score (PSS), mean (SD) | 11.92 (3.12) | 2.46 (2.34) | 5.22 (5.02) |

Abbreviations: ESAS = Edmonton Symptoms Assessment Scale; SD = Standard Deviation;

Appendix Table 2: Distribution of Individual ESAS Symptom Scores

|  | Absent  (Score: 0)  N (%) | Mild (Score: 1- 3)  N (%) | Moderate (Score: 4- 7)  N (%) | Severe (Score: 8 – 10)  N (%) | ESAS Score  Mean (SD) |
| --- | --- | --- | --- | --- | --- |
|  |  |  |  |  |  |
| Pain | 460 (22.5%) | 763 (37.4%) | 544 (26.6%) | 276 (13.5%) | 3.16 (2.69) |
| Tired | 238 (11.7%) | 667 (32.6%) | 658 (32.3%) | 480 (23.5%) | 4.15 (2.76) |
| Drowsiness | 637 (31.2%) | 681 (33.3%) | 456 (22.3%) | 269 (13.2%) | 2.79 (2.77) |
| Nausea | 1170 (57.3%) | 518 (25.4%) | 239 (11.7%) | 116 (5.7%) | 1.48 (2.31) |
| Loss of Appetite | 444 (21.7%) | 524 (25.7%) | 547 (26.8%) | 528 (25.8%) | 4.00 (3.18) |
| Shortness of Breath | 1132 (55.4%) | 566 (27.7%) | 252 (12.3%) | 93 (4.55%) | 1.46 (2.17) |
| Depression | 869 (42.5%) | 619 (30.3%) | 368 (18.0%) | 187 (9.2%) | 2.20 (2.61) |
| Anxiety | 557 (27.3%) | 702 (34.4%) | 495(24.2%) | 289 (14.2%) | 3.02 (2.79) |
| General Well-being | 245(12.0%) | 644 (31.5%) | 771 (37.7%) | 383 (18.8%) | 4.00 (2.64) |

Abbreviations: ESAS = Edmonton Symptoms Assessment Scale; SD = Standard Deviation;

Appendix Table 3: Median Survival and Hazard Ratio of individual symptoms

|  | Median Survival in months (95% CI) | Hazard Ratio*, (95% CI) | P-Value |
| --- | --- | --- | --- |
| Pain |  |  | <0.0001 |
| Absent | 8.81 (8.02, 9.76) | Ref |  |
| Mild | 7.07 (6.51, 7.63) | 1.30 (1.14, 1.47) |  |
| Moderate | 5.36 (4.77, 5.92) | 1.62 (1.42, 1.85) |  |
| Severe | 4.96 (3.95, 5.72) | 1.79 (1.52, 2.09) |  |
| Tired |  |  | <0.0001 |
| Absent | 8.84 (7.53, 10.36) | Ref |  |
| Mild | 8.42 (7.60, 9.11) | 1.17 (0.99, 1.37) |  |
| Moderate | 6.15 (5.52, 6.71) | 1.50 (1.27, 1.76) |  |
| Severe | 4.57 (3.98, 5.13) | 1.88 (1.59, 2.23) |  |
| Drowsiness |  |  | <0.0001 |
| Absent | 8.12 (7.33, 9.11) | Ref |  |
| Mild | 7.13 (6.61, 7.86) | 1.17 (1.05, 1.32) |  |
| Moderate | 5.36 (4.80, 6.25) | 1.45 (1.28, 1.65) |  |
| Severe | 3.81 (3.16, 4.57) | 1.92 (1.65, 2.24) |  |
| Nausea |  |  |  |
| Absent | 7.60 (7.10, 8.19) | Ref | <0.0001 |
| Mild | 5.85 (5.20, 6.71) | 1.30 (1.16, 1.45) |  |
| Moderate | 4.93 (3.98, 5.66) | 1.43 (1.24, 1.64) |  |
| Severe | 3.95 (2.53, 5.79) | 1.85 (1.46, 2.34) |  |
| Lack of Appetite |  |  | <0.0001 |
| Absent | 9.60 (8.48, 10.16) | Ref |  |
| Mild | 7.13 (6.51, 8.19) | 1.35 (1.17, 1.55) |  |
| Moderate | 6.18 (5.49, 6.81) | 1.47 ( 1.28, 1.69) |  |
| Severe | 4.37 (3.81, 4.87) | 2.01 (1.75, 2.30) |  |
| Shortness of Breath |  |  | <0.0001 |
| Absent | 7.40 (7.04, 7.92) | Ref |  |
| Mild | 6.51 (5.85, 7.07) | 1.14 (1.02, 1.27) |  |
| Moderate | 4.87 (3.95, 5.82) | 1.43 (1.24, 1.65) |  |
| Severe | 3.91 (2.50, 4.96) | 2.12 (1.70, 2.63) |  |
| Depression |  |  | <0.0001 |
| Absent | 7.43 (6.90, 8.19) | Ref |  |
| Mild | 6.41 (5.67, 7.10) | 1.18 (1.05, 1.31) |  |
| Moderate | 5.98 (5.46, 6.94) | 1.22 (1.07, 1.39) |  |
| Severe | 4.70 (3.75, 5.79) | 1.55 (1.31, 1.83) |  |
| Anxiety |  |  | <0.0001 |
| Absent | 7.27 (6.61, 8.15) | Ref |  |
| Mild | 7.20 (6.64, 7.92) | 1.06 (0.94, 1.20) |  |
| Moderate | 5.75 (5.26, 6.38) | 1.22 (1.07, 1.39) |  |
| Severe | 5.59 (4.96, 6.44) | 1.27 (1.09, 1.47) |  |
| Well-Being |  |  | <0.0001 |
| Best | 9.57 (8.55, 10.42) | Ref |  |
| Mild | 7.23 (6.61, 8.06) | 1.32 (1.12, 1.55) |  |
| Moderate | 6.44 (5.85, 6.94) | 1.50 (1.28, 1.76) |  |
| Worst | 4.21 (3.81, 5.00) | 1.86 (1.56, 2.22) |  |

*Hazard ratio was adjusted for age at first treatment, gender, rurality, income, Charlson’s score, previous adjuvant gemcitabine, previous pancreatic resection, and previous radiation.

Appendix Table 4: Multivariable Cox Proportional Models by Baseline ESAS Gradients

|  |  | HR | 95% CI | P - value |
| --- | --- | --- | --- | --- |
| Model 1 ^a^: |  |  |  |  |
| Baseline Total Symptom Distress Score | Severe | 1.91 | 1.45, 2.47 | <0.0001 |
|  | Moderate | 1.45 | 1.30, 1.62 | <0.0001 |
|  | Mild/Absent | Ref |  |  |
| Model 2 ^a^: |  |  |  |  |
| Baseline Physical Symptoms Scores | Severe | 1.97 | 1.53, 2.52 | <0.0001 |
|  | Moderate | 1.55 | 1.39, 1.73 | <0.0001 |
|  | Mild/Absent | Ref |  |  |
| Model 3 ^a^: |  |  |  |  |
| Baseline Psychological Symptoms Scores | Severe | 1.33 | 1.02, 1.29 | 0.02 |
|  | Moderate | 1.02 | 1.12, 1.57 | 0.001 |
|  | Mild/Absent | Ref |  |  |
| Model 4 ^a^: |  |  |  |  |
| Baseline Physical Symptoms Scores | Severe | 1.95 | 1.49, 2.55 | <0.0001 |
|  | Moderate | 1.55 | 1.38, 1.74 | <0.0001 |
|  | Mild/Absent | Ref |  |  |
| Baseline Psychological Symptoms Scores | Severe | 1.02 | 0.88, 1.12 | 0.99 |
|  | Moderate | 0.99 | 0.85, 1.23 | 1.02 |
|  | Mild/Absent | Ref |  |  |

^a^Hazard ratio was adjusted for age at first treatment, gender, rurality, income, Charlson’s score, previous adjuvant gemcitabine, previous pancreatic resection, and previous radiation.

Appendix Table 5: Sensitivity Analysis - Multivariable Cox Proportional Models on sub-cohort adjusting for ECOG status and Stage

|  |  | HR | 95% CI | P - value |
| --- | --- | --- | --- | --- |
| Model 1*: (n = 367) |  |  |  |  |
| Baseline Total Symptom Distress Score | High vs Low | 1.42 | 1.09, 1.85 | 0.009 |
| ECOG | 1+ vs 0 | 1.34 | 1.02, 1.77 | 0.04 |
| Metastasis | Metastatic vs Unresectable | 1.60 | 1.21, 2.12 | 0.001 |
| Model 2*: (n = 367) |  |  |  |  |
| Baseline Physical Symptoms Scores | High vs Low | 1.57 | 1.21, 2.10 | 0.001 |
| ECOG | 1+ vs 0 | 1.34 | 1.02, 1.77 | 0.04 |
| Metastasis | Metastatic vs Unresectable | 1.64 | 1.24, 2.17 | 0.001 |
| Model 3*: (n = 367) |  |  |  |  |
| Baseline Psychological Symptoms Scores | High vs Low | 1.08 | 0.83, 1.41 | 0.57 |
| ECOG | 1+ vs 0 | 1.36 | 1.03, 1.80 | 0.03 |
| Metastasis | Metastatic vs Unresectable | 1.61 | 1.21, 2.13 | 0.001 |
| Model 4 *: (n = 367) |  |  |  |  |
| Baseline Physical Symptoms Scores | High vs Low | 1.63 | 1.22, 2.17 | 0.001 |
| Baseline Psychological Symptoms Scores | High vs Low | 0.91 | 0.69, 1.21 | 0.53 |
| ECOG | 1+ vs 0 | 1.34 | 1.02, 1.76 | 0.04 |
| Metastasis | Metastatic vs Unresectable | 1.66 | 1.25, 2.20 | 0.0004 |

*Hazard ratio was adjusted for age at first treatment, gender, rurality, income, Charlson’s score, previous adjuvant gemcitabine, previous pancreatic resection, and previous radiation.

Appendix Table 6: Univariable median overall survival (OS) and hazard ratio (HR)

| Variable | Median OS  Days (95% CI) | HR | 95% CI | P-value |
| --- | --- | --- | --- | --- |
| ECOG (n = 367) |  |  |  | 0.002 |
| 0 | 223 (179 – 263) | Ref |  |  |
| 1+ | 168 (146 – 189) | 1.49 | 1.16 – 1.91 | 0.002 |
| Policy Regimen (n = 2043) |  |  |  | <0.0001 |
| Gem | 172 (162 – 189) | Ref |  |  |
| Folfirinox | 245 (226 – 262) | 0.76 | 0.69 – 0.84 | <0.0001 |
| Gem/nab | 166 (133 – 198) | 1.34 | 1.10 – 1.63 | 0.004 |
| Gender (n = 2043) |  |  |  | 0.12 |
| Male | 194 (180 – 210) | Ref |  |  |
| Female | 213 (195 – 226) | 0.93 | 0.85 – 1.02 | 0.12 |
| Rurality (n = 2043) |  |  |  | 0.12 |
| Rural | 176 (150 – 217) | Ref |  |  |
| Urban | 204 (193 – 215) | 0.90 | 0.79 – 1.03 | 0.13 |
| Unknown | 177 (79 – 303) | 1.28 | 0.79 – 2.10 | 0.32 |
| Income (n = 2043) |  |  |  | 0.17 |
| Highest | 221 (203 – 245) | Ref |  |  |
| Lowest | 198 (177 – 217) | 1.07 | 0.92 – 1.25 | 0.39 |
| Medium to High | 190 (168 – 210) | 1.12 | 0.97 – 1.29 | 0.13 |
| Medium to Low | 179 (163 – 201) | 1.20 | 1.03 – 1.40 | 0.02 |
| Middle | 215 (171 – 243) | 1.02 | 0.88 – 1.18 | 0.85 |
| Unknown | 232 (176 – 275) | 1.00 | 0.83 – 1.22 | 0.97 |
| Charlson (n = 2043) |  |  |  | <0.0001 |
| 0 | 198 (185 – 212) | Ref |  |  |
| 1 | 107 (77 – 134) | 1.57 | 1.16 – 2.12 | 0.003 |
| 2+ | 216 (199 – 239) | 0.86 | 0.78 – 0.95 | 0.003 |
| Previous adjuvant gemcitabine (n = 2043) |  |  |  | 0.45 |
| 0 | 201 (190 – 213) | Ref |  |  |
| 1 | 221 (179 – 297) | 0.92 | 0.76 – 1.13 | 0.45 |
| Previous Pancreatic resection (n = 2043) |  |  |  | <0.0001 |
| 0 | 189 (178 – 201) | Ref |  |  |
| 1 | 263 (226 – 295) | 0.66 | 0.59 – 0.75 | <0.0001 |
| Previous radiation  (n = 2043) |  |  |  | 0.04 |
| 0 | 208 (196 – 219) | Ref |  |  |
| 1 | 174 (148 – 199) | 1.14 | 1.003 – 1.29 | 0.04 |
| Age at first treatment  (n = 2043) |  | 1.01 | 1.005 – 1.02 | <0.0001 |

Appendix Table 7: STROBE checklist

|  | Item  No | Recommendation | Section (notes) |
| --- | --- | --- | --- |
| Title and abstract | 1 | (a) Indicate the study’s design with a commonly used term in the title or the abstract | Title (“population-base”)  Abstract (“retrospectively”, “administrative database”) |
|  |  | (b) Provide in the abstract an informative and balanced summary of what was done and what was found | Abstract: Methods and Results section |
| Introduction |  |  |  |
| Background/  rationale | 2 | Explain the scientific background and rationale for the investigation being reported | Introduction (paragraphs 1 to 5) |
| Objectives | 3 | State specific objectives, including any prespecified hypotheses | Introduction (paragraph 6) |
| Methods |  |  |  |
| Study design | 4 | Present key elements of study design early in the paper | Methods section: Study design and cohort assembly |
| Setting | 5 | Describe the setting, locations, and relevant dates, including periods of recruitment, exposure, follow-up, and data collection | Methods section:   - Study design and cohort assembly - Data sources and covariates |
| Participants | 6 | (a) Give the eligibility criteria, and the sources and methods of selection of participants. Describe methods of follow-up | Methods section:   - Study design and cohort assembly - Data sources and covariates |
|  |  | (b) For matched studies, give matching criteria and number of exposed and unexposed | N/A |
| Variables | 7 | Clearly define all outcomes, exposures, predictors, potential confounders, and effect modifiers. Give diagnostic criteria, if applicable | Methods section:   - Data sources and covariates |
| Data sources/  Measurement | 8 | For each variable of interest, give sources of data and details of methods of assessment (measurement). Describe comparability of assessment methods if there is more than one group. | Methods section:   - Data sources and covariates |
| Bias | 9 | Describe any efforts to address potential sources of bias | Methods section:   - Statistical analysis |
| Study size | 10 | Explain how the study size was arrived at | Methods section:   - Study design and cohort assembly |
| Quantitative variables | 11 | Explain how quantitative variables were handled in the analyses. If applicable, describe which groupings were chosen and why | Methods section:   - Statistical analysis |
| Statistical methods | 12 | (a) Describe all statistical methods, including those used to control for confounding | Methods   - Statistical analysis |
|  |  | (b) Describe any methods used to examine subgroups and interactions | Methods   - Statistical analysis |
|  |  | (c) Explain how missing data were addressed | Methods   - Statistical analysis |
|  |  | (d) If applicable, explain how loss to follow-up was addressed | N/A |
|  |  | (e) Describe any sensitivity analyses | Methods   - Statistical analysis |
| Results |  |  |  |
| Participants | 13 | (a) Report numbers of individuals at each stage of study—eg numbers potentially eligible, examined for eligibility, confirmed eligible, included in the study, completing follow-up, and analysed | Results   - Cohort characteristics - Figure 1 |
|  |  | (b) Give reasons for nonparticipation at each stage | Figure 1 |
|  |  | (c) Consider use of a flow diagram | Figure 1 |
| Descriptive data | 14 | (a) Give characteristics of study participants (eg demographic, clinical, social) and information on exposures and potential confounders | Results   - Cohort characteristics - Table 1 |
|  |  | (b) Indicate number of participants with missing data for each variable of interest | Table 1 |
|  |  | (c) Summarise follow-up time (eg, average and total amount) | Table 1 |
| Outcome data | 15 | Report numbers of outcome events or summary measures over time | Results   - Survival analysis - Figure 2 - Appendix Figure 1 |
| Main results | 16 | (a) Give unadjusted estimates and, if applicable, confounder-adjusted estimates and their precision (eg, 95% confidence interval). Make clear which confounders were adjusted for and why they were included | Confounders were presented in the Methods section (section “covariates))  Unadjusted and adjusted estimates were presented in the Results (section “survival analysis”)   - Table 2 (unadjusted) - Table 3 (adjusted) - Table 4 (adjusted) - Appendix Table 3 (adjusted) - Appendix Table 4(adjusted) |
|  |  | (b) Report category boundaries when continuous variables were categorized | Boundaries for continuous variables of Edmonton Symptoms Assessment Scale were described in the Methods section (section “Patient-reported outcomes- Edmonton Symptoms Assessment Scale”) |
|  |  | (c) If relevant, consider translating estimates of relative risk into absolute risk for a meaningful time period | N/A |
| Other analyses | 17 | Report other analyses done — eg analyses of subgroups and interactions, and sensitivity analyses | Results (section “survival analysis” and Appendix Table 4) |
| Discussion |  |  |  |
| Key results | 18 | Summarise key results with reference to study objectives | Discussion (paragraph 1,4) |
| Limitations | 19 | Discuss limitations of the study, taking into account sources of potential bias or imprecision. Discuss both direction and magnitude of any potential bias | Discussion (paragraph 5) |
| Interpretation | 20 | Give a cautious overall interpretation of results considering objectives, limitations, multiplicity of analyses, results from similar studies, and other relevant evidence | Discussion (paragraph 6) |
| Generalisability | 21 | Discuss the generalisability (external validity) of the study results | Discussion (paragraph 2, 3) |
| Other information |  |  |  |
| Funding | 22 | Give the source of funding and the role of the funders for the present study and, if applicable, for the original study on which the present article is based | Title page |

Appendix Table 8: Sensitivity Analysis – different methods of operationalizing ESAS scores

|  | TSDS | | PHS | | PSS | |
| --- | --- | --- | --- | --- | --- | --- |
|  | Median OS, months (95% CI) | P-value | Median OS, months (95% CI) | P-value | Median OS, months (95% CI) | P-value |
| Method 1^a^ |  |  |  |  |  |  |
| Mild | 8.0 (7.3, 8.5) | <0.0001 | 8.2 (7.3, 8.7) | <0.0001 | 7.4 (6.9, 8.2) | <0.0001 |
| Moderate | 5.8 (5.4, 6.2) |  | 5.7 (5.4, 6.3) |  | 6.3 (5.9, 6.8) |  |
| Severe | 3.9 (2.8, 4.8) |  | 4.5 (3.3, 6.0) |  | 4.5 (2.6, 5.6) |  |
| Method 2^b^ |  |  |  |  |  |  |
| Low Symptom | 7.9 (7.3, 8.4) | <0.0001 | 7.4 (7.0, 7.9) | <0.0001 | 6.9 (6.4, 7.3) | 0.0009 |
| High Symptom | 4.7 (4.1, 5.2) |  | 4.2 (3.7, 5.0) |  | 5.6 (5.0, 6.5) |  |
| Method 3^c^ |  |  |  |  |  |  |
| Low Symptom | 8.2 (7.4, 8.6) | <0.0001 | 8.2 (7.7, 8.8) | <0.0001 | 7.3 (6.9, 8.1) | <0.0001 |
| High Symptom | 5.2 (4.8, 5.6) |  | 5.0 (4.4, 5.4) |  | 5.7 (5.3, 6.2) |  |

*P-value < 0.05

^a^Method 1: For each patient, each individual symptom was categorized as mild, moderate, or severe and counted. Patients who reported majority of their individual symptoms are severe, moderate, or mild were categorized as mild, moderate, or severe, respectively. In the event of a tie, the patient is categorized as the more severe category.

^b^Method 2: For TSDS: low symptom burden is between 0 and 30; high symptom burden is between 31 – 90; For PHS: low symptom burden is between 0 and 20; high symptom burden is between 21 – 96; For PSS: low symptom burden is between 0 and 10; high symptom burden is between 11 – 20;

^b^Method 3: For each composite score (TSDS, PHS, and PSS), the mean score is calculated. Score greater than the mean were categorized as high symptom burden while score lower than the mean were categorized as low symptom burden.

Abbreviations: CI = Confidence Interval; TSDS = Baseline Total Symptom Distress Score; PHS = Baseline Physical Symptoms Scores; PSS = Baseline Psychological Symptom Scores;

Appendix Table 9: Sensitivity Analysis – different time period for collecting baseline ESAS

|  | Median Survival, months (95% CI) | |
| --- | --- | --- |
|  | High Symptom Burden | Low Symptom Burden |
| Baseline ESAS within 60 days before initiating Treatment (n = 2043) |  |  |
| TSDS | 4.64 (3.95, 5.20) | 7.46 (7.07, 8.06) |
| PHS | 4.14 (3.68, 4.80) | 7.60 (7.13, 8.12) |
| PSS | 5.56 (5.10, 6.08) | 7.10 (6.71, 7.60) |
| Baseline ESAS within 30 days before initiating Treatment (n = 1887) |  |  |
| TSDS | 4.37 (3.85, 5.00) | 7.46 (7.10, 8.09) |
| PHS | 4.08 (3.58, 4.73) | 7.53 (7.13, 8.12) |
| PSS | 5.52 (4.96, 5.98) | 7.10 (6.71, 7.56) |
| Baseline ESAS within 14 days before initiating Treatment (n = 1479) |  |  |
| TSDS | 4.37 (3.81, 5.06) | 7.76 (7.23, 8.38) |
| PHS | 3.91 (3.52, 4.70) | 7.86 (7.27, 8.42) |
| PSS | 5.36 (4.87, 5.85) | 7.36 (6.90, 7.92) |

Abbreviations: CI = Confidence Interval; TSDS = Baseline Total Symptom Distress Score; PHS = Baseline Physical Symptoms Scores; PSS = Baseline Psychological Symptom Scores;

Appendix Figure 1: Kaplan Meier Estimate of OS by Mild, Moderate and Severe Gradient of baseline composite ESAS scores

Kaplan-Meier estimates of OS by Mild, Moderate and Severe Gradient of baseline composite ESAS scores in 2,043 advanced pancreatic cancer patients who reported baseline composite ESAS scores prior to receiving first-line chemotherapy treatment. (A) Baseline composite Total Symptoms Distress Scores (TSDS), (B) Baseline composite Physical Symptoms Scores (PHS), and (C) Baseline composite Psychological Symptoms Scores (PSS).
